# The prognostic value of platelet count and lymphocyte-to-monocyte ratio, Ki-67, and Nottingham indexes in early-stage breast cancer

**DOI:** 10.1590/1806-9282.20250067

**Published:** 2025-07-07

**Authors:** Aysu Okumuş, Hüseyin Döngelli, Hüseyin Salih Semiz, Merve Keskinkılıç, Buket Hamitoğlu, Tuğba Yavuzşen

**Affiliations:** 1 Dokuz Eylul University Hospital, Department of Internal Medicine – Izmir, Türkiye.; 2 Dokuz Eylul University Hospital, Department of Medical Oncology – Izmir, Türkiye.

**Keywords:** Breast neoplasms, Hematologic tests, Prognosis, Biomarkers, Tumor, Receptors, Estrogen

## Abstract

**OBJECTIVE:**

The Nottingham Prognostic Index, Ki-67 Prognostic Index, and combination of platelet count and lymphocyte-to-monocyte ratio have shown prognostic significance in breast cancer. This aim of the study was to compare combination of platelet count and lymphocyte-to-monocyte ratio with Nottingham Prognostic Index and Ki-67 Prognostic Index in early-stage breast cancer.

**METHODS:**

A retrospective cohort study included 321 women aged ≥18 years diagnosed with early-stage breast cancer (2010–2020) who did not receive neoadjuvant chemotherapy. Data were extracted from records, with laboratory values collected preoperatively, postoperatively, and at 6-month and 1-year intervals. Regression and survival analyses evaluated the predictive value of combination of platelet count and lymphocyte-to-monocyte ratio, Nottingham Prognostic Index, and Ki-67 Prognostic Index for disease-free survival and overall survival.

**RESULTS:**

The mean age at diagnosis was 53.3 years. The mortality rate was 6.2%, and recurrence occurred in 9.3% of patients. Univariate regression showed age as the sole factor influencing overall survival (HR 1.063, p<0.001). Factors associated with disease-free survival included Luminal-B subtype (HR 3.790, p=0.029), estrogen receptor negativity (HR 15.710, p=0.007), human epidermal growth factor receptor 2 positivity (HR 4.833, p<0.001), Nottingham Prognostic Index (HR 1.967, p=0.009), and Ki-67 Prognostic Index stage (HR 1.908, p=0.008). Multivariate analysis identified age as the only significant predictor for overall survival, while human epidermal growth factor receptor 2-negativity was the sole independent factor for disease-free survival.

**CONCLUSION:**

Combination of platelet count and lymphocyte-to-monocyte ratio did not show significant prognostic value in early-stage hormone receptor-positive breast cancer. However, Nottingham Prognostic Index and Ki-67 Prognostic Index were strong predictors, particularly for disease-free survival.

## INTRODUCTION

Breast cancer is the most common cancer among women worldwide and a leading cause of cancer-related mortality^1^. Advances in early diagnosis and treatment have significantly improved prognosis and survival rates^2^. Chemotherapy sensitivity and outcomes in breast cancer are influenced by various factors, including hormone receptor (HR) status, human epidermal growth factor receptor 2 (HER2) expression, Ki-67 index, lymphovascular invasion, tumor stage, histological grade, and molecular classification^3^.

Breast cancer is categorized into non-metastatic (early and locally advanced) and metastatic stages, each with distinct prognostic features and treatment strategies. For hormone receptor-positive (HR-positive) early-stage breast cancer, treatment typically involves hormone therapy and chemotherapy^4^.

HR-positive breast cancers exhibit considerable heterogeneity in recurrence risk, with recurrences often occurring years after initial diagnosis. Factors contributing to recurrence risk include tumor grade, receptor expression, proliferative markers (e.g., Ki-67), and genomic alterations, which vary according to intrinsic subtypes such as Luminal A and B^5^. Biomarkers like estrogen receptor (ER), progesterone receptor (PR), and HER2 are critical for assessing recurrence risk and guiding therapy, with high ER/PR expression associated with a favorable response to hormone therapy^6^.

Chemotherapy influences the immune system by both suppressing and activating it against tumor cells, with immunomodulatory effects shaped by the tumor microenvironment and circulating blood cells, including platelets, lymphocytes, and monocytes^7-11^. Biomarker combinations, such as the platelet count and lymphocyte-to-monocyte ratio (COP-LMR), have shown superior prognostic value compared to individual markers^12^. COP-LMR, reflecting reactive thrombocytosis and lymphocyte-to-monocyte ratio (LMR), has emerged as a significant prognostic index in early-stage HR-positive breast cancer^13^.

The Nottingham Prognostic Index (NPI), incorporating tumor size, nodal status, and histological grade, effectively reflects metastatic behavior and tumor characteristics^14^. Similarly, the Ki-67-based Prognostic Index (KLP-PI) has demonstrated utility in predicting outcomes, particularly in the Luminal B subtype^15^.

Building on these findings, this study aims to evaluate and compare the prognostic significance of COP-LMR, NPI, and KLP-PI in early-stage HR-positive breast cancer.

## METHODS

### Patients and data collection

This retrospective cohort study included 321 female patients diagnosed with HR-positive breast cancer between 2010 and 2020. Data was collected from hospital records and the information management system.

Inclusion criteria were: pathologically confirmed HR-positive breast cancer, age ≥18 years, sufficient clinical data, and en bloc resection of the primary tumor.

Exclusion criteria were: missing data, metastatic disease at diagnosis, prior neoadjuvant chemotherapy, or male gender.

Patients who received neoadjuvant chemotherapy were excluded to avoid potential alterations in hematological parameters that could confound the prognostic value of COP-LMR.

Study variables included age at diagnosis, recurrence and mortality rates, follow-up duration, tumor size, and lymph node (LN) involvement. Luminal A subtype was defined as estrogen receptor-positive (ER-positive), progesterone receptor-positive (PR-positive) or negative, HER2-negative, and Ki-67 <14%. Luminal B included ER-positive or negative, PR-positive or negative, HER2-positive or negative, and Ki-67 ≥14%. Biomarker status was assessed using immunohistochemistry and fluorescence in situ hybridization (FISH), with HER2 (3+) considered positive, HER2 (0/1+) negative, and HER2 (2+) evaluated via FISH.

### Ethics

The study was approved by the university's Non-Interventional Research Ethics Committee (March 9, 2022; Decision No: 2022/09-12) and conducted per the Declaration of Helsinki (2000 revision).

### Laboratory examinations and indexes

Parameters were assessed preoperatively, postoperatively, and at 6 months and 1 year. COP-LMR was calculated as follows: patients with high platelet count (platelets [PLT]) and LMR were scored 2 (high risk), those with one abnormal parameter scored 1 (moderate risk), and those with neither scored 0 (low risk).

The NPI was calculated as 0.2×tumor size (cm)+grade (1–3)+LN status (0: none, 1–3: 1–3 nodes, >3: more than three nodes). Risk categories were: low (≤3.4), moderate (3.4–5.4), and high (>5.4).

The KLP-PI was calculated as 1.0×Ki-67 (≥30%: 1, <30%: 0)+1.5×LN (positive: 1, negative: 0)+1.0×R (positive: 1, negative: 0). Risk categories were low (0–1.5), moderate (2–2.5), and high (>2.5).

### Endpoints

Follow-up duration was the time from diagnosis to the last visit or death. Disease-free survival (DFS) was the time from diagnosis to recurrence, death, or last follow-up. Overall survival (OS) was the time from diagnosis to death from any cause.

### Statistical analysis

Statistical analyses were conducted using SPSS 24.0. Normality was assessed via Kolmogorov-Smirnov and Shapiro-Wilk tests. Results were expressed as n (%), mean±SD, or median (interquartile range [IQR]). Parametric variables were compared using the Friedman test.

Univariate Cox regression identified factors associated with recurrence and mortality, which were further evaluated in multivariate regression. Receiver operating characteristic (ROC) curves assessed the prognostic value of the indexes for DFS and OS. A p<0.05 was considered statistically significant.

## RESULTS

### Demographic and clinical characteristics of the patients

This retrospective cohort included 321 patients with early-stage HR-positive breast cancer who did not receive neoadjuvant chemotherapy. The mean age at diagnosis was 53.3±13.1 years, with a median follow-up of 49 months. At the end of follow-up, the mortality rate was 6.2%, and the recurrence rate was 9.3% (Table 1).

**Table 1:** Clinical characteristics of the study group.

		Total (n=321)
Age mean±SD (min–max)		53.3±13.1 (26–87)
Observation duration months median (iqr)		49 (44.5)
DFS duration months median (iqr)		46.9 (43.2)
Subtype n (%)		
	Luminal A	112 (34.9)
	Luminal B	209 (65.1)
Immunohistochemistry findings n (%)		
	Estrogen receptor	320 (99.7)
	Progesterone receptor	271 (84.4)
	HER2	48 (15)
Hormonal therapy n (%)		
	Tamoxifene	143 (44.5)
	Aromatase inhibitor agent	178 (55.5)
NPI group n (%)		
	Low	144 (44.9)
	Moderate	143 (44.5)
	High	34 (10.6)
KLP-PI n (%)		
	Low	163 (50.8)
	Moderate	122 (38.0)
	High	36 (12.0)
Preoperative COP-LMR n (%)		
	Low	90 (28.0)
	Moderate	140 (43.6)
	High	91 (28.3)
Recurrence n (%)		30 (9.3)
Death n (%)		20 (6.2)

COP-LMR: combination of platelet count and the lymphocyte/monocyte ratio; DFS: disease-free survival; HER2: human epidermal growth factor receptor 2; KLP-PI: Ki67-based prognostic index; NPI: Nottingham Prognostic Index; SD: standard deviation.

Adjuvant chemotherapy was administered to 235 patients (73.2%), and all patients received subsequent hormone therapy: 143 (44.5%) with tamoxifen and 178 (55.5%) with an aromatase inhibitor. Most patients were ER-positive (320, 99.7%), PR-positive (271, 84.4%), and HER2-negative (273, 85%).

Recurrence sites included local recurrence in nine patients (2.8%), liver metastasis in 13 (4%), bone metastasis in 12 (3.7%), brain metastasis in 1 (0.3%), distant LN metastasis in 4 (1.2%), and other organ metastases in 4 (1.2%).

### Prognostic analyses

Laboratory data were collected preoperatively, postoperatively, at 6 months, and at 1 year. Although laboratory findings showed statistically significant differences over time, no significant differences were observed between COP-LMR groups (p=0.579). Post-hoc analysis was not performed due to non-significant Friedman test results.

Univariate Cox regression analysis identified age as the sole factor significantly associated with OS (HR 1.063, p<0.001). Factors significantly associated with DFS included preoperative red blood cell (RBC), Luminal B subtype, ER negativity, HER2 positivity, NPI, and KLP-PI stage (Table 2).

**Table 2:** Univariate analysis of factors for disease-free survival and overall survival.

Variables	DFS			OS		
	HR	95%CI	p-value	HR	95%CI	p-value
Age (per one increase)	0.974	0.943–1.006	0.116	1.063	1.028–1.099	**<0.001**
WBC^a^ (per one increase)	0.847	0.690–1.040	0.112	0.893	0.700–1.140	0.363
Lymphocyte^a^ (per one increase)	0.672	0.397–1.138	0.140	0.683	0.350–1.332	0.263
Monocyte^a^ (per one increase)	0.135	0.014–1.329	0.086	1.013	0.240–4.278	0.987
RBC^a^ (per one decrease)	2.740	1.199–6.250	**0.017**	2.283	0.787–6.622	0.129
Platelet^a^ (per one increase)	0.996	0.990–1.002	0.168	0.993	0.986–1.000	0.060
Subtype (Luminal B)	3.790	1.147–12.527	**0.029**	1.161	0.419–3.216	0.775
ER negative	15.710	2.089–118.069	**0.007**	N/A	N/A	N/A
PR negative	2.044	0.873–4.787	0.099	1.814	0.599–5.493	0.292
HER2 positive	4.833	2.355–9.918	**<0.001**	1.659	0.633–4.347	0.303
NLR^a^	0.967	0.752–1.244	0.796	1.017	0.755–1.370	0.910
PLR^a^	0.998	0.992–1.005	0.640	0.998	0.989–1.007	0.694
NPI (per one stage increase)	1.967	1.180–3.277	**0.009**	1.209	0.643–2.273	0.556
KLP-PI (per one stage increase)	1.908	1.182–3.082	**0.008**	1.023	0.545–1.918	0.944
COP-LMR^a^ (per one stage increase)	0.916	0.562–1.492	0.724	0.878	0.487–1.583	0.666

CI: confidence interval; DFS: disease-free survival; HR: hazard ratio; OS: overall survival; N/A: not applicable; HER2: human epidermal growth factor receptor 2; ER: estrogen receptor; PR: progesterone receptor; COP-LMR: combination of platelet count and the lymphocyte/monocyte ratio; KLP-PI: Ki67-based prognostic index; NLR: neutrophil/lymphocyte ratio; NPI: Nottingham Prognostic Index; PLR: platelet/lymphocyte ratio; RBC: red blood cell; WBC: white blood cell.

a Preoperative values were examined.

The bold values indicate statistically significant results (p<0.05).

In multivariate analysis, age remained the only significant predictor of OS, while HER2 negativity was the sole independent factor associated with DFS (Table 3). However, when KLP-PI was treated as an ordinal variable, the high KLP-PI group showed significantly worse DFS compared to the low and intermediate groups (HR 5.302, p=0.049).

**Table 3:** Multivariate analysis of factors for disease-free survival and overall survival.

Variables	DFS			OS		
	HR	95%CI	p-value	HR	95%CI	p-value
Age (per one increase)	0.993	0.961–1.027	0.687	1.067	1.030–1.105	**<0.001**
NPI (per one stage increase)	0.959	0.454–2.024	0.912	1.285	0.555–2.679	0.558
KLP-PI (per one stage increase)	2.205	0.994–4.892	0.052	1.152	0.493–2.691	0.744
Preoperative COP-LMR (per one-stage increase)	0.888	0.527–1.495	0.654	1.028	0.556–1.902	0.926
ER negative	6.204	0.673–57.188	0.107			
PR negative	3.223	0.972–10.689	0.056			
HER2 positive	2.931	1.303–6.590	**0.009**			

CI: confidence interval; HR: hazard ratio; OS: overall survival; N/A: not applicable; COP-LMR: combination of platelet count and the lymphocyte/monocyte ratio; DFS: disease-free survival; HER2: human epidermal growth factor receptor 2; KLP-PI: Ki67-based prognostic index; NPI: Nottingham Prognostic Index; HR: hazard ratio; ER: estrogen receptor; PR: progesterone receptor.

The bold values indicate statistically significant results (p<0.05).

### Receiver operating characteristic and survival analysis

The prognostic value of KLP-PI, NPI, COP-LMR, and tumor [T], node [N], metastasis [M] (TNM) staging for DFS and OS was evaluated using ROC curves. Area under the curve (AUC) values for DFS were 0.653 (p=0.006) for KLP-PI, 0.661 (p=0.004) for NPI, 0.486 (p=0.795) for COP-LMR, and 0.632 (p=0.017) for TNM staging. For OS, the AUC values were 0.548 (p=0.474), 0.593 (p=0.164), 0.480 (p=0.760), and 0.523 (p=0.726), respectively.

## DISCUSSION

In our study, when patients were categorized into low-, moderate-, and high-risk groups based on COP-LMR values at preoperative, postoperative, 6-month, and 1-year follow-ups, no significant differences were observed between the groups or across subtypes. Among the factors evaluated for DFS, HER2 positivity, ER positivity, advanced stage, KLP-PI, and NPI stage were all found to be statistically significant. Advanced age emerged as a significant predictor of OS in both univariate and multivariate analyses. Regarding COP-LMR stages, an inverse relationship was observed in the survival curves for DFS, where the low-stage COP-LMR group exhibited a lower survival rate, contrary to existing literature^16^. However, this finding did not reach statistical significance.

LMR has been recognized as a prognostic biomarker for systemic inflammation in various malignancies, including breast cancer^11,12,17^. Previous studies have shown that low LMR is significantly associated with poorer OS in breast cancer patients^17,18^. Platelets, as reservoirs of cytokines and growth factors, play a critical role in tumor progression by promoting tumor growth and immune evasion^19^. A high preoperative platelet count has been linked to increased HER2 expression and worse prognosis in breast cancer^19^. Unlike previous studies, our research focused on early-stage HR-positive breast cancer, and the limited systemic inflammation in this cohort may explain the lack of prognostic value for LMR and platelet count, in contrast to findings in more advanced stages of the disease^11,12,19^.

When patients were categorized according to laboratory data at various time points (preoperative, postoperative, 6-month, and 1-year follow-up) into COP-LMR stages, no statistically significant differences were observed. The concept of COP-LMR staging is grounded in the hypothesis that changes in the tumor microenvironment and peripheral blood cell components influence cancer prognosis^8-11^. While one might expect to see differences in COP-LMR staging at different time points, given that all patients underwent surgery and many received adjuvant chemotherapy, our study did not reveal such differences. It is possible that advanced-stage cancer may sustain the influence of the tumor microenvironment and peripheral blood cells even after surgery or chemotherapy, while this effect may be less pronounced or absent in early-stage cancers^17,18^. In a study by Deng et al., COP-LMR demonstrated prognostic value, contrary to our findings^13^. However, in their study, COP-LMR was measured only at baseline, and it included both HR-positive and negative breast cancer patients, whereas our cohort focused exclusively on early-stage HR-positive breast cancer^13^. We believe these differences may explain the discrepancy in findings. Furthermore, in our study, we observed an inverse relationship between COP-LMR and DFS, which contrasts with previous literature^8-11,16^. While it is widely assumed that elevated inflammatory markers indicate greater tumor burden or aggressiveness, it is also plausible that a robust inflammatory response reflects a more effective host defense against tumor progression. Therefore, we interpret this finding as a hypothesis-generating observation that highlights the complexity of immune-inflammatory dynamics in cancer.

In a study by Pan et al., the prognostic role of Ki-67 in HER2-negative luminal B breast cancer was highlighted, with particular attention to the predictive value of the NPI and KLP-PI^20^. Similarly, our study demonstrated significant differences in DFS across both KLP-PI and NPI groups within the luminal A and luminal B subtypes, consistent with previous findings^20^. Notably, while the NPI effectively stratified DFS risk groups, the KLP-PI showed an even stronger prognostic impact in DFS analysis.

This study has several limitations. Although its retrospective design inherently limits causal inference and may introduce selection bias, patients with missing or incomplete data were excluded to minimize recall-related and data quality biases. Being a single-center study, our findings may have limited generalizability; thus, multicenter studies are warranted for broader validation. The exclusion of patients who received neoadjuvant chemotherapy may limit the generalizability of our findings to all early-stage breast cancer populations. Despite the valuable insights offered by retrospective studies, the low mortality and recurrence rates in our cohort limited the statistical power of survival analyses. This, along with the small sample size, may explain the lack of significant associations with OS, aside from age. Genomic assays such as Oncotype DX or MammaPrint were not included, which may limit molecular-level prognostic insights.

## CONCLUSION

Our findings suggest that NPI and KLP-PI are valuable prognostic tools for early-stage HR-positive breast cancer. In contrast, COP-LMR does not appear to have prognostic significance in this context.

## Data Availability

The datasets generated and/or analyzed during the current study are available from the corresponding author upon reasonable request.
